# Isotopic *N*,*N*-Dimethyl Leucine-Based Mass Spectrometric Quantification of Metabolites Following Copper Exposure

**DOI:** 10.3390/biom15091264

**Published:** 2025-09-01

**Authors:** Olga Riusech, Lingjun Li

**Affiliations:** 1 Department of Chemistry, University of Wisconsin-Madison, Madison, WI 53705, USA; riusech@wisc.edu; 2 Lachman Institute for Pharmaceutical Development, School of Pharmacy, University of Wisconsin-Madison, Madison, WI 53705, USA; 3 Wisconsin Center for NanoBioSystems, School of Pharmacy, University of Wisconsin-Madison, Madison, WI 53705, USA

**Keywords:** absolute quantification, hemolymph, sample preparation

## Abstract

Crustaceans are particularly sensitive to copper toxicity, and although the downstream effects of increased copper exposure on the metabolome are often postulated and observed, they are rarely measured. To perform absolute quantification of hydrophilic small-molecule metabolites in the hemolymph of the crustacean *Cancer borealis*, we derivatized targeted metabolites related to copper toxicity using in-house-developed isotopic *N*,*N*-dimethyl leucine (iDiLeu) tags. Selected analytes were pooled at previously determined concentrations to serve as internal standards, and a calibration curve was generated. The sample loss was minimized by optimizing the derivatization-assisted sample cleanup using dispersive liquid–liquid microextraction (DLLME) and hydrophilic–lipophilic balancing (HLB). Calibration curves were then used for the absolute quantification of metabolites of interest following 30 min, 1 h, and 2 h exposures to 10 µM CuCl_2_. We found that glutamic acid was downregulated after 2 h of copper exposure, which may disrupt cellular metabolism and increase oxidative stress in crustaceans. These changes could have significant impacts on crustacean populations and the ecosystems they support.

## 1. Introduction

Ocean acidification, caused by the absorption of excess atmospheric carbon dioxide (CO_2_) by seawater, is a major environmental threat that has been shown to increase the bioavailability of copper (Cu) in marine environments [1]. It is currently predicted that a drop in ocean pH of 0.3 will double the proportion of dissolved copper that is present as the free metal ion Cu^2+^—the most bioavailable form of copper [1]. This copper ion is a potent toxin that can have a range of negative effects on marine organisms. Although toxic at high concentrations, copper also plays important roles in neuromodulation, forming the structure of protein, and enzymatic activity. For example, copper is a micronutrient that is responsible for coordinating protein structures, facilitating oxygen transport, and maintaining red blood cells, nerve cells, and the immune system [2]. However, copper homeostasis is delicate; excess copper being introduced into the environment through increased ocean acidification, industrial waste, and urban runoff can lead to its accumulation in marine species [3,4,5,6]. 

The paradoxical nature of copper—as both an essential micronutrient and a potentially lethal toxin—implies the existence of sophisticated and tightly regulated biological pathways that organisms employ to manage and mitigate large environmental influxes of this metal. Dysregulated copper homeostasis has been linked to various neurological conditions, such as Alzheimer’s disease (AD) and Wilson disease [7]. While the precise role of copper in the pathogenesis of neurodegenerative diseases such as Alzheimer’s disease (AD), Parkinson’s disease (PD), and Huntington’s disease (HD) remains incompletely understood, growing evidence points to its involvement in oxidative stress and neuronal damage. Copper ions are redox-active and can catalyze the production of reactive oxygen species (ROS) and hydroxyl radicals through Fenton-like reactions. These highly reactive molecules can damage proteins, lipids, and nucleic acids, contributing to the progressive neurodegeneration observed in these disorders. In AD, a defining pathological feature is the accumulation of amyloid-beta (Aβ) plaques in the brain. These plaques have a high affinity for metal ions, particularly copper and zinc. It has been proposed that copper bound to Aβ can participate in redox cycling, generating ROS and exacerbating oxidative stress in surrounding neural tissue. This mechanism may play a critical role in the onset and progression of AD-related cognitive decline [8]. Given these observations, the identification and quantification of biomarkers associated with copper toxicity and dysregulation are of increasing interest. Such biomarkers could provide valuable insights into disease mechanisms, aid in early diagnosis, and potentially guide therapeutic strategies aimed at restoring metal homeostasis in the brain.

Small-molecule quantification offers a powerful window into the biochemical state of cells and tissues, providing critical insights into enzymatic activity, metabolic flux, and cellular signaling. These molecules, due to their dynamic and responsive nature, are strong candidates for biomarker discovery, particularly in the context of disease diagnostics and therapeutic monitoring. Among the most promising tools for small-molecule analysis is mass spectrometry (MS), which combines high sensitivity with the ability to simultaneously detect and quantify a wide array of analytes within a defined mass–charge (*m*/*z*) range. This multiplexing capability makes MS especially well-suited for untargeted and targeted metabolomics approaches aimed at identifying disease-associated metabolic signatures [9]. Many biologically relevant small molecules—including amino acids, neurotransmitters, di- and tripeptides, and certain classes of lipids—fall into the category of small, hydrophilic metabolites. However, this class of analytes presents unique analytical challenges. One major limitation is their poor ionization efficiency in electrospray ionization (ESI), the most commonly used ion source in MS-based metabolomics. Hydrophilic compounds tend to ionize less efficiently than their more hydrophobic counterparts, which results in higher limits of detection and reduced sensitivity. This discrepancy can lead to underrepresentation of key metabolites in complex biological samples, potentially skewing data interpretation [10,11]. In addition to ionization challenges, the hydrophilic nature of these metabolites complicates sample preparation and chromatographic separation. During desalting and other cleanup procedures, hydrophilic compounds are more prone to loss due to their weak interactions with solid-phase extraction materials. Furthermore, in reversed-phase liquid chromatography (RPLC)—a standard separation technique in MS workflows—these metabolites often exhibit poor retention and elute early, frequently co-eluting with salts and other matrix components that can suppress ionization or interfere with detection. These issues collectively reduce the reproducibility and reliability of quantitative measurements. To overcome these limitations, researchers have explored alternative strategies such as hydrophilic interaction liquid chromatography (HILIC), derivatization techniques to enhance ionization, and the use of internal standards tailored to specific metabolite classes. Despite these advances, the quantification of small hydrophilic molecules remains a technically demanding aspect of metabolomics [12,13,14]. 

To improve both the chromatographic retention and ionization efficiency in small-molecule mass spectrometry (MS) analysis, chemical derivatization is widely employed as a preparatory strategy. This approach involves chemically modifying analytes to enhance their physicochemical properties, which thereby improves their detectability and quantifiability [15]. A variety of derivatization reagents have been developed, each tailored to specific functional groups and analytical goals. Among the most commonly used isobaric tags for relative and absolute quantitation are iTRAQ and tandem mass tags (TMTs). These commercially available reagents offer high multiplexing capabilities, allowing simultaneous analysis of multiple samples in a single MS run. This not only increases the throughput but also enhances the signal intensity and quantification accuracy. In addition to these commercial options, our laboratory has developed a custom set of 5-plex isotopic *N*,*N*-dimethyl leucine (iDiLeu) tags. These tags are specifically designed for the absolute quantification of amine-containing compounds and offer a more cost-effective and quantitatively robust alternative to commercial reagents. The iDiLeu system enables precise quantification across multiple samples while maintaining high sensitivity and reproducibility [16,17]. However, despite the advantages of derivatization, this approach is not without limitations. The introduction of derivatization steps often necessitates additional sample cleanup and matrix simplification, which can lead to sample loss—particularly for low-abundance or labile metabolites. Moreover, incomplete derivatization or side reactions can introduce variability and complicate data interpretation. Therefore, while derivatization remains a powerful tool for enhancing MS-based small-molecule analysis, careful optimization of reaction conditions and sample handling protocols is essential to minimize artifacts and maximize analytical performance. 

A widely reported strategy to enhance small-molecule analysis in complex biological samples involves a hybridized approach that combines liquid–liquid extraction (LLE) with solid-phase extraction (SPE). This dual-step methodology has gained traction due to its effectiveness in removing interfering substances such as tagging byproducts, salts, and high-abundance lipids, all of which can compromise the sensitivity and accuracy of downstream mass spectrometry (MS) analysis [18,19]. By integrating the strengths of both extraction techniques, researchers can achieve cleaner sample matrices and improved analyte recovery, which are critical for robust and reproducible MS-based workflows. One particularly notable technique within this hybrid framework is dispersive liquid–liquid microextraction (DLLME). In DLLME, an aqueous sample is rapidly mixed with a small volume of organic solvents, typically in the presence of a dispersive solvent, which results in the formation of a fine emulsion. This process facilitates a subtle biphasic separation, allowing for efficient partitioning of target analytes into the organic phase [18,20,21]. DLLME is especially advantageous for isolating small hydrophilic molecules from complex biological matrices such as blood, plasma, or serum, where conventional extraction methods may fall short due to matrix effects or low analyte concentrations.

To further enhance selectivity and sensitivity, various workflows have been developed that integrate DLLME with chemical derivatization. These workflows vary in their timing and implementation of tagging: some protocols apply derivatization prior to DLLME to improve extraction efficiency, while others perform tagging during or after DLLME to optimize detection sensitivity. In certain cases, derivatization is omitted altogether, depending on the physicochemical properties of the analytes and the specific analytical goals of the study. Complementing DLLME, a range of SPE methods are employed to further purify and concentrate the extracted analytes. Common SPE formats include reversed-phase C18, strong cation exchange (SCX), and hydrophilic–lipophilic balance (HLB) cartridges. These sorbents offer distinct selectivity profiles, enabling researchers to obtain tailored purification strategies based on analyte characteristics. For instance, C18 and SCX have been frequently used following derivatization to exploit the increased hydrophobicity of tagged molecules, thereby enhancing the retention and enrichment. However, despite widespread use, the actual efficiency and reproducibility of these enrichment strategies have not been systematically evaluated, which highlights a need for further comparative studies to optimize extraction workflows for small-molecule analysis.

In this study, we explore matrix simplification strategies designed to enrich hydrophilic small-molecule metabolites that have been derivatized using isotopic *N*,*N*-dimethyl leucine (iDiLeu) tags. The iDiLeu labeling approach is known to significantly increase the hydrophobicity of polar metabolites, thereby altering their chromatographic behavior and extraction compatibility. Leveraging this shift in physicochemical properties, we systematically evaluate the performance of three commonly used solid-phase extraction (SPE) methods—strong cation exchange (SCX), reversed-phase C18, and hydrophilic–lipophilic balance (HLB)—to determine their efficacy in retaining and enriching iDiLeu-tagged analytes. Each method is assessed for its ability to selectively capture derivatized metabolites while minimizing background interference, with the ultimate goal of identifying the most effective matrix simplification strategy for downstream mass spectrometry (MS) analysis. To further refine the workflow, we investigate the compatibility of the optimized SPE method with dispersive liquid–liquid microextraction (DLLME), a rapid and efficient technique for isolating small molecules from complex biological matrices. DLLME is particularly attractive due to its minimal solvent requirements and high enrichment factors. By testing the SPE method both with and without DLLME, we aim to determine whether microextraction enhances or hinders the recovery of iDiLeu-labeled hydrophilic metabolites, and thereby inform the best practices for sample preparation.

To demonstrate the practical utility of our optimized workflow, we apply iDiLeu tagging to hemolymph samples collected from *Cancer borealis* (Jonah crab), focusing on the absolute quantification of five metabolites associated with copper metabolism: glycine, gamma-aminobutyric acid (GABA), glutamic acid, dopamine, and glutathione (GSH). These metabolites were selected based on their known roles in metal ion regulation, neurotransmission, and the oxidative stress response. Using a derivatization-assisted matrix simplification strategy, we incorporate isotopically labeled internal standards and implement a three-point calibration curve, reserving two iDiLeu channels for experimental samples. This multiplexed setup enables simultaneous quantification of metabolites in copper-exposed and control crab hemolymph, providing insights into the biochemical impact of copper exposure. In addition to targeted quantification, we perform label-free analysis on the same samples to identify novel metabolites that are potentially linked to copper toxicity. This untargeted approach complements the targeted workflow by expanding the metabolomic coverage and uncovering previously uncharacterized metabolic disruptions. Together, these strategies offer a comprehensive platform for studying metal-induced metabolic changes and demonstrate the versatility of iDiLeu tagging in complex biological matrices.

## 2. Materials and Methods

### 2.1. Materials 

Methanol (MeOH), acetonitrile (ACN), glacial acetic acid, and LC-MS solvents were purchased from Fisher Scientific (Pittsburgh, PA, USA). Triethylammonium bicarbonate (TEAB), *N*,*N*-dimethylformamide (DMF), 4-(4,6-dimethoxy-1,3,5-triazin-2-yl)-4-methylmorpholinium tetrafluoroborate (DMTMM), chloroform-d, and copper(II) chloride were purchased from Sigma-Aldrich (St. Louis, MO, USA). *N*-methylmorpholine (NMM) was purchased from TCI America, (Tokyo, Japan). C18 and strong cation exchange (SCX) ZipTips were purchased from Millipore (Burlington, MA, USA). Hydrophilic–lipophilic balance (HLB) columns (3cc) were purchased from Waters (Milford, MA, USA). 

### 2.2. Derivatization 

One-milligram aliquots of iDiLeu tags were obtained and dried down during activation solution preparation. Activation solution consisting of 14.08 mg DMTMM BF_4_, 495 µL dimethyl formamide, and 4.72 µL N-methyl morpholine was prepared, 50 µL of which was used to resuspend dry aliquots of iDiLeu tags. After vortexing for 1 h, 35.71 µL of activated iDiLeu tag was added to internal standard or 500 µg hemolymph previously reconstituted in 53.57 µL 0.25 M TEAB in 50% acetonitrile. Samples were tagged via vortex for 1 h at room temperature at a 1:1 m/m ratio, then quenched with 4.70 µL 5% NH_2_OH for 5 min while vortexing. Tagged samples were dried down and stored at −80 °C. 

### 2.3. Evaluating Sample Loss in SPE Methods 

C18 zip tips, SCX zip tips, and OASIS HLB columns were used for iDiLeu tag removal from aliquots of iDiLeu-tagged GABA. For C18 matrix simplification, zip tips were wetted using 50% ACN and equilibrated in 0.1% FA before loading iDiLeu-tagged GABA in 0.1% FA. Sample was then washed using 0.1% FA and eluted in water, 50% MeOH, and 100% MeOH. Elute fractions were combined. SCX was achieved similarly: loading samples in 0.1% FA, washing in 0.1% FA, and eluting in 5% NH_3_H_2_O, 30% MeOH, as previously reported. Samples desalted with HLB were resuspended in 1 mL 0.1% formic acid (FA) then pipetted onto columns previously conditioned with 1 mL MeOH and 1 mL water. Samples were subsequently rinsed with 2 mL water and eluted in 1 mL MeOH, then dried down and stored at −80 °C until LC MS/MS analysis. The flowthrough from load, wash, and elute steps from all three methods was collected and analyzed via direct infusion side-by-side with derivatized GABA without cleanup steps, representing one hundred percent signal.

### 2.4. Optimizing DLLME Workflow 

Nine aliquots of 500 µg hemolymph were used to determine if derivatization coupled with optimized desalting method would yield the highest signal in the five metabolites of interest or if DLLME could be performed before or after derivatization to enrich these small-molecule metabolites. 

DLLME protocol was completed as described: samples were resuspended in 200 µL optima-grade water and rapidly injected with 200 µL 1:10 chloroform/acetonitrile via syringe. Samples were then vortexed for 2 min and centrifuged for 5 min at 4.5 × 1000 rcf, which produced two clear layers. Supernatant was pipetted off to waste and the sediment layer was dried down and stored at −80 °C. 

### 2.5. Copper Exposure Experiments 

Male Jonah crabs, *Cancer borealis*, were purchased from local store (Global market, Madison, WI, USA) and equilibrated in tanks for one week prior to experiments. During equilibration, crabs were maintained at 11–14 °C at approximately 32 parts per thousand (ppt) salt concentration, undergoing 12 h/12 h light/dark cycles and feeding one week prior to experimentation. For each experiment, three crabs were exposed to copper (10 µM CuCl_2_) or control conditions for 30 min, 1 h, and 2 h. After incubating on ice for 30 min to anesthetize the crab, 400 µL hemolymph was collected using a syringe and directly deposited into 400 µL 90:9:1 MeOH/water/acetic acid (AcMeOH). Samples were then vortexed and centrifuged for 15 min at 18,000 rcf for protein precipitation. The supernatant was collected into weighed microcentrifuge tubes, dried down, and stored at −80 °C prior to sample preparation. For label-free analysis, sample was aliquoted and resuspended at 1 µg/µL in 0.1% FA for LC MS/MS analysis. 

**Scheme 1:**
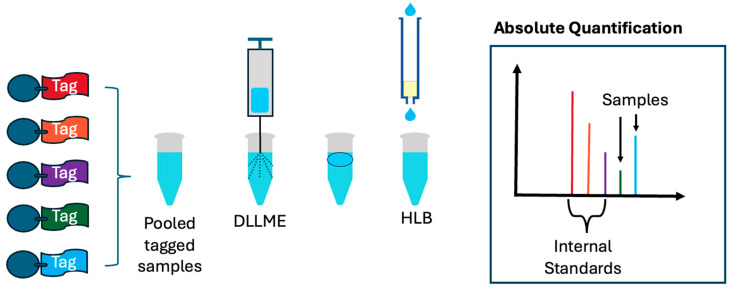
Generalized derivatization workflow.

Five hundred micrograms of hemolymph were derivatized using either d0 or d3 channels for targeted analysis of metabolites of interest. For consistent sample loss, samples were pooled with 10 µM, 1 µM, and 0.5 µM of d6, d9, and d12 channels, respectively. Pooled samples were then extracted using DLLME and desalted using HLB. Samples were then resuspended in 100 µL 0.1% FA for LC MS/MS analysis (Scheme 1). 

### 2.6. LC MS/MS 

An Ultra-High-Performance LC (UHPLC) Dionex UltiMate 3000 system equipped with a Kinetex 2.6 µm Polar C18 LC Column (100 Å, 100 × 4.6 mm) was coupled with the Q-Exactive Orbitrap mass analyzer for LC MS/MS analysis of both derivatized and label-free metabolites. All experiments utilized the same 15 min gradient with 0.1% FA as solvent A and acetonitrile with 0.1% FA as solvent B: 5–15% B from 0–7 min, 15–90% B from 7.1–10.5 min, 90% B from 10.5–12 min, 90–5% B from 12–12.5 min, and 5% B from 12.5–15 min. 

MS was performed using positive ion mode with a range of *m*/*z* of 200–500 for derivatized metabolites and a range of *m*/*z* of 70–1000 for label-free metabolites, both at a resolution of 70 K with an AGC of 10^6^ and a maximum IT of 100 ms. An inclusion list was used for all channels of iDiLeu-tagged glycine, GABA, glutamic acid, dopamine, and GSH. Data-dependent acquisition (DDA) was utilized with a collision energy of 30 eV and an isolation window of 0.5 *m*/*z*.

### 2.7. Data Analysis 

Data were processed using Xcalibur 4.4 and Compound Discoverer 3.3 (Thermo Fisher Scientific, Inc., Waltham, MA, USA). Label-free and derivatized samples were analyzed separately. For tagged samples, extracted ion chromatograms (EICs) were exported to excel from Xcalibur, where calibration curves generated from channels d6, d9, and d12 were used for the absolute quantification of samples from channels d0 and d3. Statistical significance was evaluated on excel using a Student’s *t*-test to establish which of the data were up/downregulated. Label-free data were analyzed using Compound Discoverer, where positive IDs were defined as those assigned by mzcloud. 

## 3. Results and Discussion

### 3.1. SPE Sample Loss Analysis 

**Figure 1:**
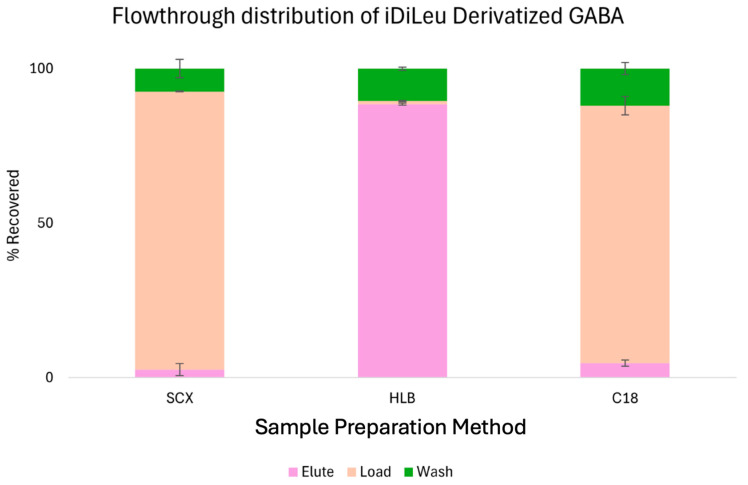
Flowthrough from elution, loading, and washing fractions of strong cation exchange (SCX), C18, and hydrophilic–lipophilic balance (HLB) solid-phase extraction (SPE) techniques repeated with three technical replicates (*n* = 3).

Although solid-phase extraction (SPE) is often bypassed in “dilute-and-shoot” workflows to minimize sample handling and potential analyte loss, it remains a critical step in derivatization-based protocols. In particular, when using isotopic *N*,*N*-dimethyl leucine (iDiLeu) tagging, SPE plays a vital role in removing excess reagents, salts, and tagging byproducts that can interfere with downstream mass spectrometry (MS) analysis. To evaluate the efficiency and potential sample loss associated with different SPE methods, we selected iDiLeu-derivatized gamma-aminobutyric acid (GABA) as a representative hydrophilic small molecule. As shown in Figure 1, three commonly used SPE sorbents—C18, strong cation exchange (SCX), and hydrophilic–lipophilic balance (HLB)—were tested for their ability to retain and recover iDiLeu-labeled GABA. During the sample loading phase, both C18 and SCX cartridges exhibited substantial analyte loss, with nearly 80% of the derivatized GABA failing to bind to the sorbent and being lost in the flowthrough. In contrast, HLB cartridges demonstrated excellent retention, with less than 3% loss during the loading step. This stark difference highlights the superior compatibility of HLB with polar, derivatized metabolites.

The wash step, designed to remove loosely bound contaminants, resulted in comparable losses across all three SPE methods, averaging around 10%. When combined with the initial loading losses, the total sample loss for C18 and SCX exceeded 90%, rendering them unsuitable for workflows involving iDiLeu-labeled hydrophilic compounds. In addition to improved analyte recovery, HLB cleanup effectively removed peaks in the mass spectrum corresponding to tagging byproducts, resulting in cleaner spectra and enhanced signal/noise ratios. Based on these findings, HLB was selected as the preferred SPE method for desalting and tag removal in our workflow. Its high retention efficiency and ability to eliminate unwanted chemical noise make it an ideal choice for derivatization-assisted metabolomics.

### 3.2. DLLME 

Dispersive liquid–liquid microextraction (DLLME) is a widely adopted sample preparation technique developed in the context of green analytical chemistry. It offers a more environmentally friendly alternative to traditional liquid–liquid extraction by significantly reducing the volume of halogenated organic solvents that is required. Unlike other microextraction methods that may involve complex apparatus or specialized reagents, DLLME is straightforward to implement and relies on solvents and equipment that are readily available in most bioanalytical laboratories. Its simplicity, speed, and cost-effectiveness make it particularly attractive for high-throughput workflows involving small-molecule analysis.

**Figure 2:**
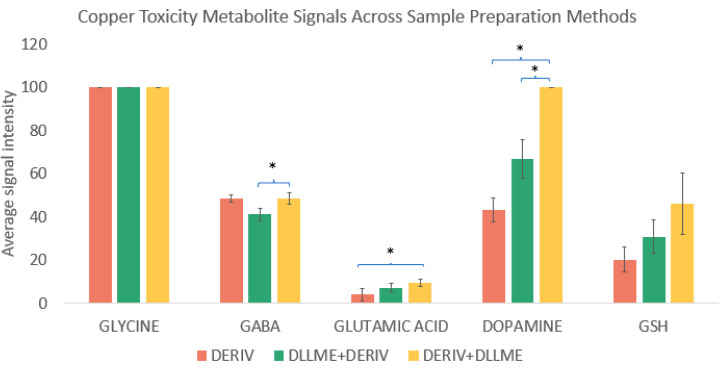
Signal intensities of the five metabolites of interest across three technical replicates (*n* = 3)—glycine, gamma aminobutyric acid (GABA), glutamic acid, dopamine, and glutathione (GSH). Statistical significance of *p* < 0.05 is indicated by (*).

In this study, we evaluated the impact of DLLME on the recovery and enrichment of iDiLeu-derivatized hydrophilic metabolites. Specifically, we compared three protocols: the standard workflow involving derivatization followed by desalting, DLLME performed prior to derivatization, and DLLME performed after derivatization. The results, summarized in Figure 2, reveal distinct differences in metabolite behavior depending on the timing of DLLME.

Glycine, a small and highly polar amino acid, showed no significant change in recovery across the three protocols, which indicated that DLLME neither enriched nor depleted its concentration. However, glutamic acid and dopamine exhibited statistically significant enrichment when DLLME was applied after derivatization. This suggests that the increased hydrophobicity imparted by iDiLeu tagging enhances the partitioning of these molecules into the organic phase during DLLME, improving their recovery. Notably, both GABA and dopamine showed greater enrichment when DLLME was performed after derivatization compared to before. This observation supports the hypothesis that derivatization alters the physicochemical properties of these metabolites in a way that favors their extraction via DLLME. Based on these findings, we adopted the post-derivatization DLLME protocol for subsequent experiments, as it provided superior enrichment for key metabolites of interest. This optimized workflow—combining iDiLeu tagging with post-derivatization DLLME—offers a robust and efficient strategy for enhancing the detection and quantification of hydrophilic small molecules in complex biological matrices.

### 3.3. Targeted Quantification Results 

**Figure 3:**
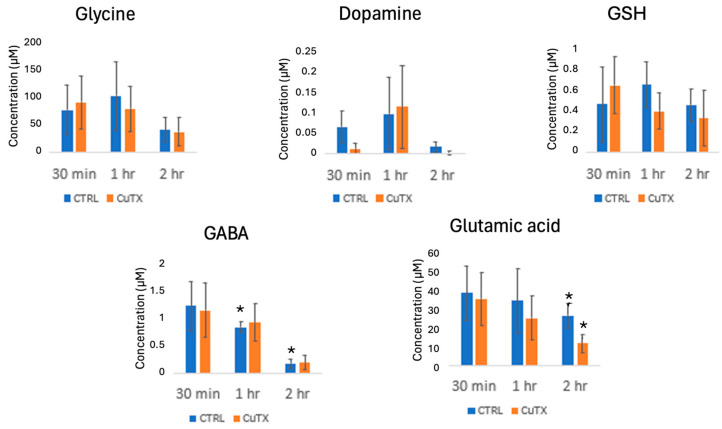
Absolute concentrations of glycine, dopamine, glutathione (GSH), gamma aminobutyric acid (GABA), and glutamic acid are reported across three crab samples (*n* = 3). GABA concentration is shown to steadily decrease over time, while glutamic acid is downregulated at the 2 h mark in copper-exposed crabs. Statistical significance of *p* < 0.05 is indicated by (*).

Analytes were successfully detected and quantified down to concentrations as low as 10 nM, with dopamine serving as a representative low-abundance metabolite, as shown in Figure 3. This level of sensitivity highlights the robustness of the optimized workflow, which combines iDiLeu derivatization, solid-phase extraction (SPE), and post-derivatization DLLME. The ability to detect such low concentrations is particularly important when working with hemolymph samples, where metabolite levels can vary widely depending on the physiological and environmental conditions.

To assess the impact of copper exposure on metabolite levels, hemolymph samples from Jonah crabs were analyzed following 30 min, 1 h, and 2 h exposure periods. No statistically significant changes were observed in the 30 min and 1 h groups, which suggests that short-term exposure may not be sufficient to elicit measurable metabolic responses. However, in the 2 h exposure group, a notable downregulation of gamma-aminobutyric acid (GABA) was observed in control crabs between 1 h and 2 h of exposure, possibly due to stress or dietary depletion over the duration of the experiment. However, more in-depth mechanistic studies are required to understand the role of GABA in this context.

Glutamic acid, another key metabolite, plays a dual role in copper metabolism. It has been proposed as a potential antioxidant treatment for copper toxicity and serves as a precursor in the biosynthesis of glutathione (GSH), a tripeptide that is critical for cellular defense against oxidative stress [22]. GSH binds copper ions and maintains them in a reduced, non-toxic state, thereby protecting cells from copper-induced cytotoxicity [23]. In our study, glutamic acid was significantly downregulated in crabs exposed to copper for 2 h, with the average concentrations falling to 11.8 µM. This reduction suggests that glutamic acid is being rapidly consumed for GSH synthesis, which potentially outpaces its replenishment. Given that glutathione plays a central role in copper transport and detoxification [2], the observed depletion of glutamic acid may indicate a metabolic bottleneck. If the glutamic acid availability continues to decline, it could limit future GSH synthesis, exacerbating oxidative stress and impairing copper homeostasis. These findings align with previous research suggesting that copper-induced oxidative damage may be driven, in part, by glutathione depletion [24], which underscores the importance of monitoring precursor metabolites in metal toxicity studies. 

### 3.4. Label-Free Results 

Label-free analysis was performed to complement the targeted quantification of copper-related metabolites and to explore broader metabolic changes induced by copper exposure. Volcano plots generated using Compound Discoverer revealed the differential regulation of several hundred compounds across experimental conditions. These plots highlighted both upregulated and downregulated features, providing a global view of metabolic perturbations. However, only eight compounds were confidently identified through positive MS/MS spectral matches supported by the mzCloud database. Notably, all eight identified compounds were significantly downregulated under copper-toxic conditions. The confirmed downregulated compounds include nortriptyline, 2-(cyclohexylmethylidene)-1,2,3,4-tetrahydronaphthalen-1-one, taurine, (2E)-3-[(1R,4S,7R,7aR)-1-hydroxy-3,7-dimethyl-2,4,5,6,7,7a-hexahydro-1H-inden-4-yl]-2-methylprop-2-enoic acid, prolylleucine, leucylproline, 5-methoxyindole, and amobarbital. 

Among these, taurine stands out due to its well-documented role in metal detoxification and antioxidant defense. Taurine is a sulfonated amino acid known to influence drug and metal absorption indirectly through its antioxidant properties [25,26,27,28]. Prior studies have shown that dietary taurine supplementation can enhance copper excretion and protect against copper-induced nephrotoxicity in mouse models [26,27]. In mussel hemolymph, taurine downregulation has also been linked to glutamic acid depletion, which suggests a coordinated response to metal stress [26]. While the other downregulated compounds are less commonly associated with copper toxicity, nortriptyline—a tricyclic antidepressant—has been reported to exhibit neuroprotective effects by preventing calcium ion (Ca^2+^) overload [29,30]. This mechanism is reminiscent of mitochondrial taurine, which also contributes to Ca^2+^ homeostasis. The concurrent downregulation of taurine and nortriptyline may reflect a disruption in calcium regulation pathways under copper stress, which potentially contributes to neurochemical imbalance or oxidative damage. The remaining compounds, including prolylleucine, leucylproline, and 5-methoxyindole, may represent novel biomarkers of copper toxicity. Although their roles are not yet fully understood, their consistent downregulation warrants further investigation. Overall, these label-free results provide valuable insights into the broader metabolic consequences of copper exposure and highlight potential targets for future mechanistic studies. 

## 4. Conclusions

Metabolomics profiling of crustacean hemolymph provides a powerful lens through which to examine both cellular and systemic biochemical responses to environmental stressors, particularly heavy metal exposure. Hemolymph, the circulatory fluid in crustaceans, reflects dynamic physiological changes and serves as an accessible matrix for monitoring metabolic perturbations. Mass spectrometry (MS)-based metabolomics enables the simultaneous detection and quantification of a broad spectrum of metabolites within a single sample, offering both high sensitivity and comprehensive coverage. This dual capability is especially valuable in ecotoxicological studies, where subtle shifts in metabolite levels can signal early biological responses to toxicants such as copper.

In this study, we employed a combined analytical strategy that incorporated both iDiLeu-based chemical tagging and label-free MS approaches to investigate metabolic changes associated with copper toxicity in Jonah crab, *Cancer borealis*. The iDiLeu derivatization method was particularly advantageous for enhancing the detection of hydrophilic metabolites, which are often poorly retained in conventional workflows. To maximize the analyte recovery and signal intensity, we optimized the sample preparation protocol by performing derivatization prior to desalting. This sequence improved retention of polar compounds during solid-phase extraction and minimized sample loss.

Our time-course exposure experiments revealed that short-term copper exposure (30 min and 1 h) did not result in statistically significant global metabolic shifts. However, targeted analysis revealed a consistent decrease in glutamic acid levels, even at these early time points. This observation suggests that glutamic acid may serve as an early biomarker of copper-induced metabolic stress. Given its role as a precursor in glutathione biosynthesis and its involvement in neurotransmission, glutamic acid depletion may have downstream consequences, including impaired antioxidant defense and altered neurochemical balance. Interestingly, this early reduction in glutamic acid may be mechanistically linked to the observed downregulation of taurine in the label-free analyses. Taurine has been implicated in modulating glutamate levels and maintaining cellular redox balance, particularly under metal stress. The coordinated decline in these metabolites points to a potential early disruption in amino acid metabolism and antioxidant pathways.

Future studies involving longer exposure durations, higher copper concentrations, or recovery time points may reveal more pronounced dysregulation in copper-associated metabolic pathways. Such investigations could further elucidate the temporal dynamics of copper toxicity and identify additional biomarkers for environmental monitoring and crustacean health assessment.

## Data Availability

The raw data supporting the conclusions of this article will be made available by the authors upon request.
